# Standardized High-Quality Processes for End-of-Life-Decision Making in the Intensive Care Unit Remain Robust during an Unprecedented New Pandemic—A Single-Center Experience

**DOI:** 10.3390/ijerph192215015

**Published:** 2022-11-15

**Authors:** Fanny Marsch, Claudia D. Spies, Roland C. E. Francis, Jan A. Graw

**Affiliations:** 1 Department of Anaesthesiology and Operative Intensive Care Medicine (CCM, CVK), Charité—Universitätsmedizin Berlin, Augustenburger Platz 1, 13353 Berlin, Germany; 2 Department of Anesthesiology, Universitätsklinikum Erlangen, Friedrich-Alexander-Universität Erlangen-Nürnberg, 91054 Erlangen, Germany; 3 Department of Anesthesiology and Intensive Care Medicine, Universitätsklinikum Ulm, Ulm University, 89081 Ulm, Germany

**Keywords:** end-of-life decision, advance directive, intensive care unit, shared decision making, COVID-19

## Abstract

Due to the global COVID-19 pandemic, a concomitant increase in awareness for end-of-life decisions (EOLDs) and advance care planning has been noted. Whether the dynamic pandemic situation impacted EOLD-processes on the intensive care unit (ICU) and patient-sided advance care planning in Germany is unknown. This is a retrospective analysis of all deceased patients of surgical ICUs of a university medical center from March 2020 to July 2021. All included ICUs had established standardized protocols and documentation for EOLD-related aspects of ICU therapy. The frequency of EOLDs and advance directives and the process of EOLDs were analyzed (No. of ethical approval EA2/308/20). A total number of 319 (85.5%) of all deceased patients received an EOLD. Advance directives were possessed by 83 (22.3%) of the patients and a precautionary power of attorney by 92 (24.7%) of the patients. There was no difference in the frequency of EOLDs and patient-sided advance care planning between patients with COVID-19 and non-COVID-19 patients. In addition, no differences in frequencies of do-not-resuscitate orders, withholding or withdrawing of intensive care medicine therapeutic approaches, timing of EOLDs, and participation of senior ICU attendings in EOLDs were noted between patients with COVID-19 and non-COVID-19 patients. Documentation of family conferences occurred more often in deceased patients with COVID-19 compared to non-COVID-19 patients (COVID-19: 80.0% vs. non-COVID-19: 56.8, *p* = 0.001). Frequency of EOLDs and completion rates of advance directives remained unchanged during the pandemic compared to pre-pandemic years. The EOLD process did not differ between patients with COVID-19 and non-COVID-19 patients. Institutional standard procedures might contribute to support the robustness of EOLD-making processes during unprecedented medical emergencies, such as new pandemic diseases.

## 1. Introduction

While death is common in the intensive care unit (ICU), most ICU patients in the Western world die after an end-of-life decision (EOLD) [1]. An EOLD describes a decision to limit ICU therapeutic approaches with regard to medical futility, the patient’s will, or both. Due to the severity of the disease, most patients cannot actively participate in an EOLD [2]. In recent years, many countries have established a legal basis for advance directives and advance care planning [3]. With an advance directive or advance care planning, patients can preserve their autonomy in the moments that they are losing decision-making capacity due to a serious illness or injury. Promotion for completion of advance directives by legal reforms and informal support might have led to an increase of advance directive completion rates over the last decade [4,5,6]. However, completion rates of advance directives remain low in general [7,8].

In early 2020, the COVID-19 pandemic spread around the world, causing an unprecedented impact on health care systems and societies [9]. As a new and unknown infection, early reports from China described an airborne, highly contagious virus disease causing respiratory failure potentially worsening to an acute respiratory distress syndrome (ARDS) with considerable mortality [10,11,12]. Healthcare systems were therefore challenged and sometimes overwhelmed by the extreme large amount of patients with respiratory failure and ARDS requiring mechanical ventilation [12,13,14].

The public awareness on the COVID-19-associated mortality, especially among the elder population, was substantial. As indicated by recent data from social media platforms, the COVID-19 pandemic led to a considerable increase of communication about ICU-specific life-sustaining treatments and advance care planning [15]. It is unclear whether the public awareness might have triggered potential early discussions of advance care planning. However, in several countries, admissions to the ICU were withheld based on limitation decisions that were made before hospital admission [16]. Reflections about life-sustaining treatment and advance care planning are often affected by emotional sentiments. Discrimination of elderly and disabled people through EOLDs was intensely discussed in public [15]. In the light of ICU resources potentially not meeting the demand for ventilators, equipment, and healthcare personnel, the fear for undifferentiated end-of-life decision making and triage increased [17].

The aim of the current study was to evaluate whether the unprecedented pandemic situation had an impact on EOLD processes in a large ICU of a German tertiary care university hospital treating both patients with COVID-19 respiratory failure and non-COVID-19 critically ill patients. Furthermore, the presence of forms of advance care planning among deceased ICU patients was analyzed. In addition, EOLD processes and advance care planning were compared between patients admitted with COVID-19 and non-COVID-19 patients.

## 2. Materials and Methods

### 2.1. Study Design and Setting

This retrospective cohort study includes all patients who died between 1 March 2020 and 31 July 2021 in the surgical ICUs of the Department of Anesthesiology and Intensive Care Medicine at Charité—Universitätsmedizin Berlin, encompassing a total of 51 ICU beds. Due to the ongoing COVID-19 pandemic, ICUs included specialized COVID-19 ARDS-ICUs, non-COVID-19 ICUs, and ICUs with mixed patient populations, where COVID-19 patients were separated from non-COVID-19 patients in special isolation rooms. During the peaks of the COVID-19 infection waves, the department was running a specialized COVID-19 ARDS-ICU in newly built, high-level ICU facilities on the hospital’s campus, with a total of 31 beds. Capacity was dynamically adjusted to the national infection process and according to the forecast for expected ICU resources in the state.

Due to the visitor restrictions applied for the ICUs treating COVID-19 patients, appointments for patient relatives with the treating ICU consultants and family conferences were scheduled by ICU case managers and ICU-associated psychologists and conducted by phone or videoconference. Consultants with board certification in intensive care medicine led the daily rounds and were available 24/7. At least once a day, consultant specialist surgeons from the surgical disciplines admitting patients to the ICU were present in ward rounds. There was continuous in-house coverage by consultants board-certified in Anesthesiology and Intensive Care Medicine. Furthermore, there were Intensive Care Medicine fellows and Anesthesia residents continuously present on each ICU. The Department of Anesthesiology and Intensive Care Medicine at Charité—Universitätsmedizin Berlin has an established quality-management program and is certified according DIN EN ISO 9001. Medical standard of care is provided with the support of defined and regularly reevaluated standard operating procedures for ICU processes and procedures, including EOLD making. An EOLD, a do-not-resuscitate (DNR) order, and an order to withhold and/or withdraw life support (WH/WDLS) were defined as described previously [3]. Patients received an EOLD only when every participant of the EOLD conference consented to the decision and the individual patient regulations. The study was approved by the ethical committee of Charité—Universitätsmedizin Berlin (EA2/308/20).

### 2.2. Data Sources

Data on patients’ demographics were extracted from the hospital data management system (SAP, Walldorf, Germany). Data on admission scores, comorbidities, ICU treatment and organ replacement therapies, code status, advanced care planning, and progress notes including family communication, EOLD conferences, processes, and communication were extracted from the electronic intensive care unit data-management system (COPRA 6, Sasbachwalden, Germany) as described previously [3]. The acute physiology and chronic health evaluation II (APACHE II) and the simplified acute physiology score II (SAPS II) were calculated automatically by the PDMS.

### 2.3. Statistical Analysis

Results are expressed as arithmetic mean ± standard deviation (SD) for continuous variables and frequencies (%) for categorical variables, respectively. Due to the sample sizes and the skewness of distributions, only nonparametric (exact) tests were applied. Differences between groups were tested by the nonparametric (exact) Wilcoxon–Mann–Whitney test for independent groups. Frequencies were tested by the (exact) chi-square test. A two-tailed *p*-value < 0.05 was considered statistically significant. All tests were conducted in the area of exploratory data analysis. Therefore, no adjustments for multiple testing were made. All numerical calculations were performed with statistical software package SPSS^®^ Version 28 (IBM, Armonk, North Castle, NY, USA).

## 3. Results

During the observation period, 4402 patients were admitted to the ICUs, of whom 373 died (8.4%). There were 248 patients with COVID-19 admitted to the ICUs, with considerably higher mortality (29.0%). Epidemiologic data and patient baseline parameters are shown in Table 1. Patients dying with COVID-19 were younger, more frequently male, and had a longer ICU-LOS compared to non-COVID-19 patients (Table 1). Deceased patients admitted with COVID-19 compared to non-COVID-19 patients did not differ in baseline comorbidities except for a higher rate of metastatic cancer in non-COVID-19 patients (Table 2). There were only few patients with COVID-19 admitted perioperatively compared to perioperative admissions of non-COVID-19 patients (COVID-19: 9.8% vs. non-COVID-19: 45.1%, *p* < 0.001).

A total number of 319 (85.5%) of all deceased patients received an EOLD, with no difference in the frequency of EOLDs between patients with COVID-19 and non-COVID-19 patients (COVID-19: 83.3% vs. non-COVID-19: 86.0%, *p* = 0.577). Advance directives were possessed by 83 (22.3%) of the deceased patients and a precautionary power of attorney by 92 (24.7%) patients, again with no differences in frequencies between patients with COVID-19 and non-COVID-19 patients (Advance directive: COVID-19: 20.8% vs. non-COVID-19: 22.6%, *p* = 0.757; precautionary power of attorney: COVID-19: 24.7% vs. non-COVID-19: 23.6%, *p* = 0.880). The frequency of documentation of EOLD-relevant information about patient-sided advance directives and a precautionary power of attorney in the special section of the PDMS was higher in deceased patients with COVID-19 compared to deceased non-COVID-19 patients (COVID-19: 73.6% vs. non-COVID-19: 37.5%, *p* < 0.001).

Taken together, these data suggest that the frequency of EOLDs and the number of patients possessing an advance directive or a precautionary power of attorney remained similar to pre-pandemic years. Most importantly, the frequency of EOLDs did not differ between patients with COVID-19 and non-COVID-19 patients.

The frequencies of comorbidities in patients that received an EOLD did not differ between COVID-19 and non-COVID-19 patients (Table 2).

During their course on the ICU, patients with COVID-19 were more often tracheotomized and were treated more often with ECMO or renal replacement therapy (RRT) compared to non-COVID patients (Table 3). At the time of the EOLD, patients with COVID-19 were more often on RRT compared to non-COVID patients. However, both groups did not differ in the frequency of treatment with mechanical ventilation at the time of the EOLD (Table 3).

Details on the EOLD process in the subgroup of deceased patients with a preceding EOLD are reported in Table 4.

The frequency of patients with a DNR order and a WHLS order did not differ between patients with COVID-19 and non-COVID-19 patients (Table 4). Patients with COVID-19 received more often a successive decision from DNR to WH/WDLS (COVID-19: 13.3% vs. non-COVID-19: 5.4%, difference: 7.9%, *p* = 0.044), while the frequency of multi-step approaches for WH/WDLS decisions did not statistically differ between both groups (COVID-19: 63.3% vs. non-COVID-19: 52.5%, *p* = 0.151, Table 4). EOLDs were done 24/7, with the majority of decisions made during the core working hours between 7 a.m. and 4 p.m. (Table 4). Shifts when EOLDs were made did not differ between patients with COVID-19 and non-COVID-19 patients. The mean number of days from ICU admission to the first EOLD was higher in patients with COVID-19 compared to non-COVID-19 patients (COVID-19: 18.14 ± 23.37 days vs. non-COVID-19: 6.53 ± 8.48 days, *p* < 0.001). In nine out of ten EOLDs, one or more senior consultants with an ICU board certification were documented as responsibly leading the decision-making process. Fellows board-certified in anesthesia and intensive care medicine were more often present in EOLDs of patients with COVID-19 compared to EOLDs in non-COVID 19 patients (Table 4).

Patient participation and patient information in EOLDs was low in both patients with COVID-19 and non-COVID-19 patients (Table 5). The patient’s family or surrogate decision maker in the EOLD process was almost always informed, and frequencies did not differ between patients with COVID-19 and non-COVID-19 patients (COVID-19: 90.0% vs. non-COVID-19: 88.0%, *p* = 0.824, Table 5). However, participation of the patient’s family or surrogate decision maker in the EOLD process occurred more often in non-COVID-19 patients compared to patients with COVID-19 (COVID-19: 51.7% vs. non-COVID-19: 67.2%, difference: 15.5%, *p* = 0.026, Table 5). The frequency of documentation on details of the EOLD in the special section of the PDMS did not differ between deceased patients with COVID-19 compared to deceased non-COVID-19 patients (Table 5). However, the content of family conferences was more often documented in the special section of the PDMS in patients with COVID-19 (Table 5). Similar to data reported for the whole cohort, documentation of legal documents such as patient-sided advance directives and a precautionary power of attorney was more frequently done in deceased patients with COVID-19 compared to deceased non-COVID-19 patients (Table 5).

Taken together, these data suggest that the core process of EOLD making remained similar between deceased patients with COVID-19 and non-COVID-19 patients. Although family members and surrogate decision makers were less often involved in the EOLD process, intensive and detailed communication in form of family conferences occurred more frequently in patients with COVID-19.

## 4. Discussion

In the investigated cohort of ICU patients, the frequency of EOLDs and completion rates of advance directives remained unchanged during the pandemic compared to pre-pandemic years. The EOLD process did not differ between patients with COVID-19 and non-COVID-19 patients. However, documentation of family conferences occurred more often in patients with COVID-19 compared to non-COVID-19 patients, while participation of the patients’ family or surrogate decision maker in the EOLD process occurred less often in patients with COVID-19 compared to non-COVID-19 patients.

Although increasing over the recent decade, completion rates of advance directives remain low in Germany [6]. Seniors seem to complete an advance directive more frequently compared to people in the working age [8]. However, there is also evidence that older people and patients with progressive comorbidities have thought about end-of life issues within their families and selected substitute decision makers, but most of them did not complete written documents or discuss their preferences with a physician [18]. Data from web-based advance directive platforms indicated that during the COVID-19 pandemic, a significant increase in the demand for advance directive documentation was noted [19]. The completion rates of advance directives and legal documents among ICU patients reported in this study are similar to completion rates reported to corresponding patient collectives prior to the COVID-19 pandemic [6]. However, documentation of EOLD-relevant information about patient-sided advance care planning increased, especially in ICU patients admitted with COVID-19, compared to previous years and in comparison to data reported from other European centers [6,16].

With the pictures of overwhelmed ICU capacities in Italy or Spain with the first wave of the COVID-19 pandemic in 2020, options to save a maximum number of lives were waged in each country. Besides increasing the number of ICU beds and organizing intelligent transfer concepts to also dynamically include distant ICUs, two ethical problematic options came up for discussion: prioritization of ICU beds for patients with the best prognosis and accelerated withdrawal of life support in the ICU once necessary [16]. As a matter of fact, and due to a nationwide central steering of ICU resources in Germany, there was always sufficient ICU capacity for COVID-19 and non-COVID-19 patients in Germany [20,21]. However, certain nervousness among healthcare workers regarding a potential loss of control in the situation was noticeable [22]. Furthermore, public fear for a decision-making shift from a patient-centered to a population-centered approach with discrimination of elderly or disabled was articulated [15]. Here, we report from a large patient cohort of deceased ICU patients during multiple waves of the COVID-19 pandemic that the frequency of EOLDs and key aspects of the decision-making process did not differ between patients with COVID-19 compared to non-COVID-19 patients. Like reported from multiple other studies, deceased patients with COVID-19 were more frequently male, had a longer ICU-LOS, and received more ICU organ replacement and support therapy than non-COVID-19 patients [23,24]. High-quality end-of-life practice is associated with ICU end-of-life protocols and country end-of-life legislation [25]. Like many other European countries, German legislation guides and supports EOLD processes and the use of advance care planning [1,5]. As stated above, in the ICUs of the study center, EOLD and end-of-life practice was performed according to departmental standard operating procedures and protocols [3,26]. It is likely that decision making based on institutional standard operating procedures and quality-management measures ensured continuous and established decision making and care processes for healthcare workers and patients even during unprecedented medical emergencies, such as a new pandemic disease. Moreover, during the peaks of the COVID-19 waves in Germany, an additional 100 ICU beds were established at our hospital. Staffing included multidisciplinary and multi-professional medical teams from different medical and surgical backgrounds under the lead of physicians board-certified for anesthesiology and intensive care medicine. Establishing a work environment based on standard operating procedures and protocols guaranties not only made workflow and patient care more efficient but also medical treatment, according to the current evidence and standard of care [27,28].

Interestingly, although the patient’s family and surrogate decision makers were informed about a patient’s EOLD, participation of the family and surrogate decision makers in the EOLD process was lower in patients with COVID-19 compared to non-COVID-19 patients. Based on the retrospective nature of the study, reasons for this finding remain speculative; however, during the observation period, strict visitor restrictions for ICUs treating patients with COVID-19 were instituted by hospital policies. For families, perception of the course of a patient’s disease was only possible by regular phone calls and intermittent video calls and conferences. However, increased use of digital resources and communication tools facilitating communication with families may have contributed to the increased documentation of the content of family conferences. EOLD-associated legal documentation also increased and was significantly higher in patients with COVID-19. Whether increased documentation efforts were due to increased awareness of palliative care or legal aspects in patients with COVID-19 or due to increased research interest and data collection from patients with COVID-19 in a university hospital remains speculative [29]. A similar explanation might account for the higher participation frequency of board-certified anesthesia and intensive care medicine fellows in EOLDs of patients with COVID-19. The documentation efforts showed also significantly higher rates compared to the already increased documentation efforts of EOLD-associated documentation from pre-pandemic years [6]. This finding was underlined by data showing that during treatment of patients with COVID-19, effective remote communication of the health care team, the patient, and the patient’s family was associated with significantly better ratings of the overall experience of end-of-life care by the patient’s family members [30].

Limitations of the current study include the retrospective and single-center design with an exploratory data analysis approach due to the number of studied patients. As stated previously, there was never a prioritization of ICU care necessary in Germany during the COVID-19 pandemic. However, it is unclear if preemptive code status discussion or decisions to avoid advanced ICU therapy in individual patients might have impacted admissions to the ICU during the pandemic waves. Whether EOLD processes remain robust in a setting with significantly limited ICU resources remains unclear. Only deceased patients were included in the study and due to the ongoing pandemic, and many operations requiring ICU resources postoperatively were postponed. Therefore, the patient cohort for non-COVID-19 patients shows a shifting to mainly emergency admissions, with consecutive inhomogeneity with classical non-COVID-19 ICU patient populations.

## 5. Conclusions

Taken together, these data obtained from a decent cohort of deceased ICU patients during the first 1.5 years of the COVID-19 pandemic in Germany demonstrated that the frequency of EOLDs and completion rates of advance directives remained unchanged during the pandemic compared to pre-pandemic years. In addition, the EOLD process did not differ between patients with COVID-19 and non-COVID-19 patients. Institutional standard procedures might contribute to the robustness of EOLD-making processes during unprecedented medical emergencies, such as new pandemic diseases.

## Figures and Tables

**Table 1:** Characteristics of all deceased patients.

	All (*n* = 373)		COVID-19 (*n* = 72)		Non-COVID-19 (*n* = 301)		*p*-Value
Age, years, mean (±SD)	68.88	(15.13)	65.85	(±13.68)	69.61	(±15.39)	0.015
Gender, male, *n* (%)	218	(58.4)	54	(75.0)	164	(54.5)	0.002
Urgency of admission, *n* (%)							
Elective	10	(2.7)	-	-	10	(3.3)	<0.001
Unplanned	26	(7.0)	15	(20.8)	11	(3.7)	
Emergency	337	(90.3)	57	(79.2)	280	(93.0)	
Severity scores, mean (±SD)							
APACHE II	31.24	(8.88)	30.49	(±9.08)	31.42	(±8.84)	0.479
SAPS2	69.14	(18.65)	70.97	(±17.84)	68.70	(±18.85)	0.174
ICU-LOS, days, mean (±SD)	10.35	(14.36)	19.35	(±22.35)	8.20	(±10.65)	<0.001
EOLD, *n* (%)	319	(85.5)	60	(83.3)	259	(86.0)	0.577
Advance directive, *n* (%)	83	(22.3)	15	(20.8)	68	(22.6)	0.757
Precautionary power of attorney, *n* (%)	92	(24.7)	17	(23.6)	75	(24.9)	0.880

**Table 2:** Patient Comorbidities.

Comorbidity, *n* (%)	All (*n* = 373)		COVID-19 (*n* = 72)		Non-COVID-19 (*n* = 301)		*p*-Value
Liver cirrhosis	28	(7.5)	4	(5.6)	24	(8.0)	0.622
Portal hypertension	13	(3.5)	3	(4.2)	10	(3.3)	0.722
Status post esophageal bleeding	5	(1.3)	1	(1.4)	4	(1.3)	1.000
Hepatic encephalopathy	5	(1.3)	1	(1.4)	4	(1.3)	1.000
Heart failure NYHA IV	31	(8.3)	4	(5.6)	24	(9.0)	0.477
Chronic pulmonary disease	72	(19.3)	13	(18.1)	59	(19.6)	0.869
Chronic obstructive pulmonary disease (COPD)	56	(15.0)	6	(8.3)	50	(16.6)	0.097
Lung fibrosis	8	(2.1)	1	(1.4)	7	(2.3)	1.000
Terminal renal insufficiency	16	(4.3)	1	(1.4)	15	(5.0)	0.327
Steroid medication	15	(4.0)	2	(2.8)	13	(4.3)	0.745
Chemotherapy	11	(2.9)	0	(-)	11	(3.7)	0.133
Immunosuppression therapy	15	(4.0)	3	(4.2)	12	(4.0)	1.000
HIV-Infection status positive	0	(-)	0	(-)	0	(-)	-
Leukemia	11	(2.9)	0	(-)	11	(3.7)	0.133
Lymphoma	10	(2.7)	3	(4.2)	7	(2.3)	0.414
Metastatic cancer	41	(11.0)	2	(2.8)	39	(13.0)	0.011

**Table 3:** ICU Therapies.

ICU Therapies, *n* (%)	All (*n* = 373)		COVID-19 (*n* = 72)		Non-COVID-19 (*n* = 301)		*p*-Value
Tracheostomy	53	(14.2)	23	(31.9)	30	(10.0)	<0.001
Mechanical ventilation	347	(93.0)	71	(98.6)	276	(91.7)	0.038
ECMO Therapy	56	(15.0)	27	(37.5)	29	(9.6)	<0.001
Renal replacement therapy	129	(34.6)	43	(59.7)	86	(28.6)	<0.001
Mechanical ventilation at EOLD	259	(69.4)	54	(75.0)	205	(68.1)	0.260
Renal replacement therapy at EOLD	93	(24.9)	30	(41.7)	63	(20.9)	<0.001

**Table 4:** EOLD process in patients deceased with EOLD.

EOLD Process, *n* (%)	All (*n* = 319)		COVID-19 (*n* = 60)		Non-COVID-19 (*n* = 259)		*p*-Value
DNR	284	(89.0)	51	(85.0)	233	(90.0)	0.358
WH/WDLS	292	(91.5)	54	(90.0)	238	(91.6)	0.797
Advance directive	82	(25.7)	15	(25.0)	67	(25.9)	1.000
Precautionary power of attorney	88	(27.6)	16	(26.7)	72	(27.8)	0.875
Successive decision from DNR to WH/WDLS	22	(6.9)	8	(13.3)	14	(5.4)	0.044
Multi-step approach for WH/WDLS	174	(54.6)	38	(63.3)	136	(52.5)	0.151
*Shift of EOLD, n (%)*							
Core working time	177	(55.5)	31	(51.7)	146	(56.4)	0.585
Late shift	106	(33.2)	20	(33.3)	86	(33.2)	
Night shift	36	(11.3)	9	(15.0)	27	(10.4)	
*Physicians responsible in EOLD, n (%)*							
Senior Attending	288	(90.3)	54	(90.0)	234	(90.3)	1.000
Junior Attending/Fellow	57	(17.9)	26	(43.3)	31	(12.0)	<0.001
Resident	83	(26.0)	14	(23.3)	69	(26.6)	0.629
Attending-associated specialty	77	(24.1)	12	(20.0)	65	(25.1)	0.504

**Table 5:** EOLD communication and documentation in patients deceased with EOLD.

EOLD Communication, *n* (%)	All (*n* = 319)		COVID-19 (*n* = 60)		Non-COVID-19 (*n* = 259)		*p*-Value
Patient participated in EOLD	17	(5.3)	1	(1.7)	16	(6.2)	0.475
Patient was informed of EOLD	13	(4.1)	1	(1.7)	12	(4.6)	0.213
Family/Surrogate decision maker participated in EOLD	205	(64.3)	31	(51.7)	174	(67.2)	0.026
Family/Surrogate decision maker was informed of EOLD	282	(88.4)	54	(90.0)	228	(88.0)	0.824
*Documentation in PDMS* *special section, n (%)*							
Legal documents	149	(46.7)	44	(73.3)	105	(40.5)	<0.001
Content of family conferences	195	(61.1)	48	(80.0)	147	(56.8)	0.001
Detailed EOLD process	201	(63.0)	42	(70.0)	159	(61.4)	0.237

## Data Availability

Data are available from the corresponding author on reasonable request.
